# Trends and clusters of tuberculosis treatment interruption among people experiencing homelessness in Brazil: influence of individual, social and programmatic factors

**DOI:** 10.1590/1980-549720250041

**Published:** 2025-08-08

**Authors:** Gabriel Pavinati, Lucas Vinícius de Lima, Melisane Regina Lima Ferreira, Simone Teresinha Protti Zanatta, Gabriela Tavares Magnabosco

**Affiliations:** I Universidade Estadual de Maringá, Graduate Program in Nursing - Maringá (PR), Brazil.; II Secretaria Municipal de Saúde, Gerência de Planejamento - Maringá (PR), Brazil.; III Ministério da Saúde, Coordenação-Geral de Vigilância da Tuberculose, Micoses Endêmicas e Micobactérias Não Tuberculosas - Brasília (DF), Brazil.; IV Universidade Federal de São Carlos, Graduate Program in Nursing - São Carlos (SP), Brazil.

**Keywords:** Tuberculosis, Ill-housed persons, Treatment adherence and compliance, Health status disparities, Ecological studies

## Abstract

**Objective:**

To analyze temporal trends and state-level clusters of tuberculosis treatment interruption indicators among the homeless population in Brazil.

**Methods:**

This is an ecological study, in which treatment interruption among homeless people with tuberculosis was assessed from 2015 to 2023. Joinpoint regression was used for trend analysis, stratified by sociodemographic and epidemiological variables. State clusters were identified by *k*-means clustering analysis, based on socioeconomic and programmatic indicators.

**Results:**

Tuberculosis treatment interruption increased among: men (average quarterly percent change - AQPC=0.15; 95% confidence interval - 95%CI 0.04-0.29), individuals aged 40-59 years (AQPC=0.38; 95%CI 0.25-0.53), tobacco users (AQPC=0.72; 95%CI 0.61-0.82), beneficiaries of social programs (AQPC=4.59; 95%CI 3.69-6.02), those without directly observed treatment (AQPC=0.49; 95%CI 0.39-0.63), without HIV coinfection (AQPC=0.38; 95%CI 0.30-0.51), and in the North (AQPC=1.51; 95%CI 0.96-2.21) and Midwest (AQPC=0.83; 95%CI 0.17-1.59) regions. According to the cluster analysis, cluster A had the lowest treatment interruption rate, low AIDS incidence, and better programmatic indicators. Cluster B had high poverty and low level of education and income, but strong primary health care performance. Cluster C stood out for its higher human development, better social indicators, and lower inequality. Cluster D concentrated the worst outcomes: higher treatment interruption, greater inequality, higher AIDS incidence, and weaker primary health care.

**Conclusion:**

Socioeconomic and programmatic inequalities were evident in access and attachment to tuberculosis treatment among people experiencing homelessness in Brazil.

## INTRODUCTION

The close relationship between socioeconomic aspects and the global tuberculosis epidemic underlines the importance of incorporating the Sustainable Development Goals (SDGs) in tackling the disease^1^. According to the World Health Organization (WHO), seven of these goals are directly linked to the prevalence of tuberculosis, in addition to goal three, which aims to eliminate it by covering areas such as reducing inequalities, poverty, and hunger, among other topics^1^.

The investigation of the occurrence of tuberculosis, an aggravation of strong social determination, in people experiencing homelessness (PEH) has been the subject of several studies. The highest burden of tuberculosis in PEH was reported in research conducted in different countries, among them: Korea^2^, Ethiopia^3^, India^4^ , United States of America and Brazil^5^ ^,^ ^6^. It is understood that the extreme social fragility faced by this invisible population is decisive in the maintenance of this public health issue.

Globally, an estimated total of 10.8 million people fell ill from tuberculosis in 2023, equivalent to 134 new cases per 100 thousand inhabitants^1^. In Brazil, only in 2024, 84,308 new cases of tuberculosis were officially recorded, representing an incidence rate of 39 cases per 100 thousand inhabitants^7^. Of this number, it is estimated that 3.6% were cases among people experiencing homelessness, being the second vulnerable group most affected by the disease^7^.

Ambitious targets were proposed, nationally and internationally, to eliminate tuberculosis as a public health issue by 2035^1^ ^,^ ^2^ ^,^ ^3^ ^,^ ^4^ ^,^ ^5^ ^,^ ^6^ ^,^ ^7^. However, obstacles persist in the access and attachment of the affected persons to healthcare services, causing a relevant phenomenon, especially among PEH: treatment interruption^6^ ^,^ ^8^ ^,^ ^9^. In Brazil, researchers showed that the cure of tuberculosis among homeless people may be less than 50% in some states, while the interruption may exceed 40%^8^.

Different factors may explain the increased susceptibility of PEH to treatment interruption, such as: age reduction, pulmonary tuberculosis, HIV coinfection, and drug use^5^. They can, however, be minimized with interventions such as directly observed treatment (DOT) and financial aid^5^. Nevertheless, the temporal behavior of tuberculosis cure indicators in Brazil is worrisome: decreasing trends have been reported in most states^10^.

Tuberculosis, as a marker of social inequalities, poses additional challenges to PEH. In addition to the structural barriers faced by this population, such as lack of housing and food insecurity, issues related to stigmatization and discrimination also contribute to the exclusion from healthcare services. All of this intensifies the difficulties of effective implementation of treatment, requiring intersectoral interventions focused on the reduction of inequalities.

By understanding the temporal behavior of tuberculosis treatment interruption in PEH, considering potential factors influencing adherence and attachment, and the areas most vulnerable to its occurrence, through socioeconomic and programmatic aspects, this study may be crucial to guide strategies based on the SDGs. Therefore, we analyzed the temporal trends and state-level clusters of indicators of tuberculosis treatment interruption among PEH in Brazil.

## METHODS

This is an ecological study reported on the basis of the Reporting of Studies Conducted using Observational Routinely-Collected Health Data. The focus was on the evaluation of the indicators of tuberculosis treatment interruption in PEH, by time series and nonhierarchical clustering analyses. Brazil was the study scenario, a country politically and administratively divided into 27 federative units (FUs), contemplated by 450 health regions and 5,570 municipalities and districts.

The population of PEH is defined as a heterogeneous group, which has extreme poverty, interrupted or weakened family bonds, and the absence of regular conventional housing in common^11^. According to data from the Brazilian Ministry of Human Rights and Citizenship, in 2022, there were 236,400 people experiencing homelessness, with the proper registration in the Single Registry for Social Programs (*Cadastro Único para Programas Sociais*), that is, one out of one thousand people in the country were homeless^11^.

For this study, data were extracted from the Notifiable Diseases Information System (*Sistema de Informação de Agravos de Notificação* - SINAN) on August 4, 2024, in the “*Transferência de Arquivos*” [File Transfer] section, and tabulated by TabWin (https://datasus.saude.gov.br/transferencia-de-arquivos/). All cases in which the variables of interest were blank or filled as “ignored” were disregarded, assuming that this information would not allow adequate and reliable analysis of the notified cases.

Cases with the variable “person experiencing homelessness” filled as “yes” were included. For the time frame, the period from 2015 to 2023 was determined, as variables for special populations (i.e., PEH) were inserted in the notification form in 2015. The inclusion criteria were: adults, considered those aged ≥18 years, who had started the treatment for tuberculosis as a new case and who had ended it by interrupting the treatment (i.e., abandonment).

As new cases, those with the entry types “new case” and “do not know” were considered, similar to the definition established by the Brazilian Ministry of Health^11^. Treatment interruption was considered all cases of tuberculosis that had “abandonment” or “primary abandonment” as end of treatment status. For delimiting the year of registration, the variable “year of diagnosis” was considered; and, for the referral to the place of health care, the variable “notification FU” was used.

The development of the indicator of interruption of treatment for tuberculosis in PEH was based on the report of the *Caderno de Indicadores da Tuberculose* [Journal of Tuberculosis Indicators]^12^ of the Ministry of Health. The calculation was as follows: division of the number of new cases with the outcome of treatment interruption in a given period and population (numerator) by the total number of new cases recorded in the same period and with the same population (denominator), and the result was multiplied by 100.

For trend analysis, the joinpoint method was used, allowing the location of inflection points in the series^13^. The analyses were stratified by: notification region (North; Northeast; Southeast; South; Midwest); sex (men; women); age group (in years: 18-39; 40-59; ≥60 or over); beneficiary of social programs (yes; no); HIV (positive; negative); use of drugs (yes; no), tobacco (yes; no), or alcohol (yes; no); diabetes (yes; no); and DOT (yes; no).

For the analysis of the characteristics of individuals who interrupted treatment, a common denominator was considered, corresponding to the total number of interruptions recorded in each year. This approach aimed to identify changes in the composition of this outcome over time, allowing to observe which subgroups became more representative. It is not a risk estimate, but rather a descriptive strategy aimed at understanding the predominant profile among cases of interruption.

As time series analysis presupposes a minimum number of eight points for trends to be appropriately estimated^13^, quarterly indicators were used, totaling 36 periods. However, as a reduced number of events could generate significant random variations in percentage measurement^12^, the three-point moving average strategy for smoothing series was employed, which resulted in series with 34 points (considered as smoothed quarters).

The quarterly indicators of treatment interruption for tuberculosis were transformed by natural logarithmic function due to better interpretation and comparison of results. The log-linear models were adjusted by the standard percentage errors and by the first-order autocorrelation estimated from the data^13^. The final models, estimated by grid research, were chosen by the lowest value of the weighted Bayesian information criterion^13^.

For each final model, the following were calculated: quarterly percent change (QPC) regarding the change of indicators in each joinpoint; average quarterly percent change (AQPC) related to the geometric averages of QPC; and 95% confidence intervals (95%CI). In the interpretation, positive QPC/AQPC indicated an increasing trend, while negative QPC/AQPC, a decreasing trend. QPC/AQPC whose 95%CI did not include the null value were considered significant.

In addition, the analysis of nonhierarchical clustering was performed by the *k*-means method, which divides the dataset into *k* groups, reducing the sum of square distances between points and their respective centroids. It is assumed that the data within each group (*k*) are more similar to each other than to data from other groups, with the distance between points and centroids as parameter. For this study, the *k* value was defined by the elbow method.

The 26 Brazilian states and the Federal District were defined as units of analysis. The variables deemed mediators in the clustering were selected according to the theoretical proximity to the study outcome - tuberculosis treatment interruption -, according to previous studies^5^ ^,^ ^14^ ^,^ ^15^. The detailed description of each indicator is shown in Chart 1. The variables of each group were described as a function of arithmetic mean and standard deviation (SD).

**Chart 1.** State indicators from the Brazilian Institute of Geography and Statistics (IBGE), the Brazilian Health Informatics Department (DATASUS), and the Health Information System for Primary Health Care (SISAB).

Indicator	Description
Human Development Index	Assessment of long-term progress in three fundamental dimensions: long and healthy life, access to knowledge, and decent living standards. The final indicators are consolidated on a scale ranging from 0.00 to 1.00, where a value closer to 1.00 indicates a better overall development.
Gini Index	Measurement of the degree of inequality in the distribution of per capita household income. The value of the index ranges from 0.00 to 1.00, where 0.00 represents perfect equality (everyone has the same income) and 1.00 represents perfect inequality (one person has all the income while others have nothing).
Percentage of those vulnerable to poverty	Number of individuals with per capita household income equal to or less than BRL 255.00 per month, divided by the total population; the result was multiplied by 100.
Percentage of poor people	Number of individuals with per capita household income equal to or less than BRL 140.00 per month, divided by the total population; the result was multiplied by 100.
Percentage of people in extreme poverty	Number of individuals with per capita household income equal to or less than BRL 70.00 per month, divided by the total population; the result was multiplied by 100.
Final synthetic indicator	The final synthetic indicator of the *Previne Brasil* [Brazil Prevents] Program is the final calculation of a set of seven primary health care performance indicators. Its value ranges from zero to ten, being obtained from the assignment of the individual score for each indicator and the weighting by their respective weights.
Acquired immunodeficiency syndrome incidence rate	Number of newly-diagnosed cases of acquired immunodeficiency syndrome (codes B20 to B24 of the International Classification of Diseases - 10th edition) recorded in the considered year, divided by the total population; the result was multiplied by 100 thousand.
Percentage of hospitalizations due to conditions sensitive to primary health care	Percentage of hospitalizations due to conditions sensitive to primary health care (according to the list of the International Classification of Diseases - 10th edition), in relation to the total number of clinical admissions, in a given geographical space and year.
Illiteracy rate	Population aged 18 years or older who cannot read or write a simple note, divided by the total number of people in this age group; the result was multiplied by 100.
Per capita income	Sum of income (in BRL) of all individuals living in permanent private households, divided by the total number of these individuals.

The groups obtained by the *k*-means clustering analysis were statistically compared using the Kruskal-Wallis test, which verifies if there are significant differences in the distribution of a variable between three or more independent groups from the ranks of the values. In the presence of significance, that is, p≤0.050, the Mann-Whitney test was performed for peer-to-peer comparisons, assessing whether the distributions of two groups differ between each other, without assuming normality of the data.

For the analyses, the Joinpoint Regression Program^®^ (version 5.0.2) and SPSS Statistics^®^ (version 25.0) were used. It should be noted that this study is part of the doctoral dissertation *Desafios na continuidade do tratamento da tuberculose na população em situação de rua: proteção social e padrões ecológicos no contexto brasileiro*, presented to the Graduate Program in Nursing of Universidade Estadual de Maringá. Approval was obtained from the Research Ethics Committee (Opinion No. 6.736.365/2024).

### Data availability statement

The entire dataset that supports the results of this study was made available on Mendeley Data and can be accessed at https://doi.org/10.17632/zfypkvyhg3.1.

## RESULTS

**Figure 1.**
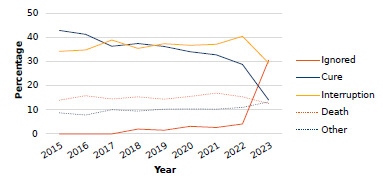
Annual percentages of the outcomes of tuberculosis treatment in the homeless population, according to cure, treatment interruption, death, ignored, and other outcome, Brazil, 2015-2023.

From 2015 to 2023, 18,474 cases of tuberculosis were recorded among PEH in Brazil, with variation in treatment outcomes over the years (Figure 1). Of this number, 5,981 (32.38%) people evolved with the cure of the disease, 2,747 (14.87%) died, 1,920 (10.39%) had other outcomes (such as bankruptcy, transfer, etc.), 1,188 (6.43%) had no record of end of treatment, and 6,638 (35.93%) interrupted the tuberculosis treatment - this being the universe of cases of the present study.

Regarding the characteristics, there was a predominance of treatment interruption among men, aged 18 to 39 years, who used alcohol, tobacco and illicit drugs, and who were located in the Southeast region. Over the years, the absence of DOT and the receipt of benefits from social programs prevailed. Most did not present diabetes or HIV; however, it is noteworthy that the occurrence of tuberculosis-HIV coinfection exceeded 20% of cases in all years (Table 1).

**Table 1.** Annual percentages of the interruption of tuberculosis treatment among people experiencing homelessness according to demographic, socioeconomic, and epidemiological characteristics, Brazil, 2015-2023.

Variable	Period								
	2015	2016	2017	2018	2019	2020	2021	2022	2023
Sex									
Men	79.15	80.28	81.67	83.33	81.72	83.24	81.72	82.66	83.42
Women	20.85	19.72	18.33	16.67	18.28	16.76	18.28	17.34	16.58
Age group (years)									
18-39	59.22	58.98	60.28	59.64	56.54	55.80	52.69	55.15	55.25
40-59	36.50	37.73	35.86	36.71	39.20	40.68	43.71	40.53	40.55
≥60	4.28	3.29	3.86	3.65	4.26	3.52	3.60	4.32	4.20
Directly observed treatment									
Yes	35.37	32.78	31.40	29.25	30.76	26.29	25.11	27.70	25.49
No	64.63	67.22	68.60	70.75	69.24	73.71	74.89	72.30	74.51
Diabetes									
Yes	3.35	2.81	3.97	3.21	3.03	3.15	2.89	2.99	3.77
No	96.65	97.19	96.03	96.79	96.97	96.85	97.11	97.01	96.23
Use of alcohol									
Yes	52.31	55.34	53.27	52.96	52.26	54.85	56.25	57.05	54.53
No	47.69	44.66	46.73	47.04	47.74	45.15	43.75	42.95	45.47
Use of tobacco									
Yes	45.86	46.64	49.85	49.59	52.42	53.68	51.60	56.81	57.31
No	54.14	53.36	50.15	50.41	47.58	46.32	48.40	43.19	42.69
Use of drugs									
Yes	57.91	59.92	62.19	64.19	62.35	63.77	66.16	65.64	65.28
No	42.09	40.08	37.81	35.81	37.65	36.23	33.84	34.36	34.72
Beneficiary of social programs									
Yes	4.43	7.12	8.13	9.85	8.59	10.37	9.81	11.92	15.35
No	95.57	92.88	91.87	90.15	91.41	89.63	90.19	88.08	84.65
HIV coinfection									
Yes	28.39	26.37	29.83	30.68	25.98	23.84	23.10	22.61	21.48
No	71.61	73.63	70.17	69.32	74.02	76.16	76.90	77.39	78.52
Notification region									
North	3.96	4.63	7.05	6.11	9.88	7.74	8.71	9.86	7.72
Northeast	11.71	13.46	15.60	11.96	12.25	11.38	11.35	11.61	9.98
Southeast	60.45	63.12	58.74	59.63	57.54	60.67	58.96	57.02	62.25
South	19.62	14.88	14.48	16.76	16.48	15.10	15.58	16.81	14.04
Midwest	4.26	3.91	4.13	5.54	3.85	5.11	5.40	4.70	6.01

There was an increasing trend of the interruption of tuberculosis treatment for: men (AQPC=0.15; 95%CI 0.04-0.29), 40-59 years (AQPC=0.38; 95%CI 0.25-0.53), absence of DOT (AQPC=0.49; 95%CI 0.39-0.63), tobacco use (AQPC=0.72; 95%CI 0.61-0.82), beneficiary of social programs (AQPC=4.59; 95%CI 3.69-6.02), absence of HIV coinfection (AQPC=0.38; 95%CI 0.30-0.51), and the North (AQPC=1.51; 95%CI 0.96-2.21) and Midwest (AQPC=0.83; 95%CI 0.17-1.59) regions, as shown in Table 2.

**Table 2.** Temporal trend of the percentages of tuberculosis treatment interruption among people experiencing homelessness, according to demographic, socioeconomic, and epidemiological characteristics, Brazil, 2015-2023.

Variable	Quarter (Year)	QPC (95%CI)	AQPC (95%CI)
Sex			
Men	Q2 (2015)-Q2 (2018)	0.39 (0.14-2.39)*	0.15 (0.04-0.29)*
	Q2 (2018)-Q3 (2023)	0.01 (-0.87-0.11)	
Women	Q2 (2015)-Q4 (2018)	-1.97 (-2.78 to -1.41)*	-0.62 (-0.90 to -0.36)*
	Q4 (2018)-Q4 (2019)	4.15 (0.83-7.79)*	
	Q4 (2019)-Q3 (2020)	-5.87 (-8.68 to -1.66)*	
	Q3 (2020)-Q1 (2022)	3.62 (2.40-8.88)*	
	Q1 (2022)-Q4 (2022)	-8.73 (-11.39 to -4.78)*	
	Q4 (2022)-Q3 (2023)	5.25 (1.40-13.49)*	
Age group (years)			
18-39	Q2 (2015)-Q1 (2018)	0.18 (-0.19-0.88)	-0.15 (-0.26 to -0.02)*
	Q1 (2018)-Q3 (2021)	-0.90 (-2.53 to -0.63)*	
	Q3 (2021)-Q3 (2023)	0.70 (0.09-2.00)*	
40-59	Q2 (2015)-Q3 (2016)	1.31 (0.38-3.38)*	0.38 (0.25-0.53)*
	Q3 (2016)-Q3 (2017)	-2.31 (-4.04 to -0.77)*	
	Q3 (2017)-Q3 (2021)	1.45 (1.17-1.79)*	
	Q3 (2021)-Q3 (2022)	-2.46 (-4.03-1.36)	
	Q3 (2022)-Q3 (2023)	0.57 (-1.00-3.23)	
≥60	Q2 (2015)-Q3 (2023)	0.41 (-0.08-0.95)	0.41 (-0.08-0.95)
Directly observed treatment			
Yes	Q2 (2015)-Q3 (2023)	-0.94 (-1.23 to -0.66)*	-0.94 (-1.23 to -0.66)*
No	Q2 (2015)-Q2 (2018)	1.05 (0.73-1.56)*	0.49 (0.39-0.63)*
	Q2 (2018)-Q1 (2019)	-2.89 (-3.96 to -0.37)*	
	Q1 (2019)-Q4 (2020)	2.14 (1.36-4.26)*	
	Q4 (2020)-Q2 (2022)	-1.17 (-2.93 to -0.29)*	
	Q2 (2022)-Q3 (2023)	0.96 (0.04-0.63)*	
Diabetes			
Yes	Q2 (2015)-Q3 (2023)	-0.01 (-1.04-1.19)	-0.01 (-1.04-1.19)
No	Q2 (2015)-Q3 (2023)	0.00 (-0.04-0.04)	0.00 (-0.04-0.04)
Use of alcohol			
Yes	Q2 (2015)-Q4 (2016)	1.33 (0.46-4.03)*	0.05 (-0.14-0.26)
	Q4 (2016)-Q4 (2017)	-2.18 (-3.89 to -0.41)*	
	Q4 (2017)-Q4 (2022)	0.57 (0.43-0.93)*	
	Q4 (2022)-Q3 (2023)	-2.96 (-7.03 to -0.72)*	
No	Q2 (2015)-Q4 (2016)	-1.58 (-4.75 to -0.39)*	-0.06 (-0.29-0.14)
	Q4 (2016)-Q4 (2017)	2.71 (0.66-5.10)*	
	Q4 (2017)-Q4 (2022)	-0.69 (-1.23 to -0.51)*	
	Q4 (2022)-Q3 (2023)	3.63 (0.54-8.11)*	
Use of tobacco			
Yes	Q2 (2015)-Q2 (2020)	0.92 (0.76-1.13)*	0.72 (0.61-0.82)*
	Q2 (2020)-Q3 (2021)	-1.36 (-3.96 to -0.33)*	
	Q3 (2021)-Q2 (2022)	4.28 (2.19-5.68)*	
	Q2 (2022)-Q3 (2023)	-0.05 (-1.76-0.68)	
No	Q2 (2015)-Q2 (2020)	-0.90 (-1.12 to -0.73)*	-0.77 (-0.89 to -0.64)*
	Q2 (2020)-Q3 (2021)	1.40 (-0.07-3.84)	
	Q3 (2021)-Q2 (2022)	-4.78 (-6.16 to -2.27)*	
	Q2 (2022)-Q3 (2023)	0.03 (-1.20-2.86)	
Use of drugs			
Yes	Q2 (2015)-Q3 (2018)	0.97 (0.80-1.24)*	0.37 (0.31-0.45)*
	Q3 (2018)-Q2 (2019)	-1.87 (-2.55 to -0.39)*	
	Q2 (2019)-Q3 (2021)	0.93 (0.65-2.12)*	
	Q3 (2021)-Q3 (2023)	-0.36 (-0.81 to -0.05)*	
No	Q2 (2015)-Q3 (2018)	-1.42 (-1.94 to -1.09)*	-0.58 (-0.70 to -0.45)*
	Q3 (2018)-Q4 (2019)	1.59 (0.27-4.00)*	
	Q4 (2019)-Q3 (2021)	-2.10 (-4.92 to -1.30)*	
	Q3 (2021)-Q3 (2023)	0.79 (0.13-2.15)*	
Beneficiary of social programs			
Yes	Q2 (2015)-Q4 (2016)	15.02 (6.84-34.50)*	4.59 (3.69-6.02)*
	Q4 (2016)-Q4 (2021)	0.69 (-2.51-1.63)	
	Q4 (2021)-Q3 (2023)	7.48 (3.93-15.39)*	
No	Q2 (2015)-Q4 (2016)	-0.90 (-1.90 to -0.48)*	-0.45 (-0.55 to -0.38)*
	Q4 (2016)-Q1 (2022)	-0.09 (-0.18-0.13)	
	Q1 (2022)-Q3 (2023)	-1.25 (-2.36 to -0.71)*	
HIV coinfection			
Yes	Q2 (2015)-Q1 (2016)	-5.53 (-10.63 to -1.63)*	-1.09 (-1.34 to -0.87)*
	Q1 (2016)-Q2 (2018)	2.80 (1.89-4.57)*	
	Q2 (2018)-Q1 (2020)	-5.11 (-7.24-1.31)	
	Q1 (2020)-Q4 (2020)	3.02 (-5.19-4.99)	
	Q4 (2020)-Q3 (2023)	-1.47 (-3.67 to -0.81)*	
No	Q2 (2015)-Q1 (2016)	2.58 (1.18-4.94)*	0.38 (0.30-0.51)*
	Q1 (2016)-Q1 (2018)	-1.35 (-2.33 to -0.93)*	
	Q1 (2018)-Q1 (2020)	1.72 (-0.50-2.59)	
	Q1 (2020)-Q4 (2020)	-0.86 (-1.39-2.27)	
	Q4 (2020)-Q3 (2023)	0.44 (0.16-1.19)*	
Notification region			
North	Q2 (2015)-Q1 (2016)	-10.20 (-24.62 to -0.26)*	1.51 (0.96-2.21)*
	Q1 (2016)-Q1 (2017)	23.78 (15.93-36.61)*	
	Q1 (2017)-Q3 (2018)	-4.35 (-9.90 to -1.49)*	
	Q3 (2018)-Q3 (2019)	18.62 (12.90-26.78)*	
	Q3 (2019)-Q3 (2020)	-11.56 (-17.09 to -6.81)*	
	Q3 (2020)-Q2 (2022)	6.00 (4.09-10.53)*	
	Q2 (2022)-Q3 (2023)	-7.11 (-11.09 to -4.35)*	
Northeast	Q2 (2015)-Q2 (2017)	5.50 (3.21-10.12)*	-0.04 (-0.45-0.59)
	Q2 (2017)-Q1 (2018)	-9.29 (-12.42 to -1.51)*	
	Q1 (2018)-Q3 (2023)	-0.67 (-1.30-1.30)	
Southeast	Q2 (2015)-Q2 (2022)	-0.26 (-0.96 to -0.04)*	0.08 (-0.22-0.32)
	Q2 (2022)-Q3 (2023)	2.01 (-0.08-6.85)	
South	Q2 (2015)-Q4 (2016)	-7.39 (-10.51 to -5.11)*	-1.47 (-1.88 to -1.12)*
	Q4 (2016)-Q3 (2018)	3.75 (1.59-9.67)*	
	Q3 (2018)-Q4 (2021)	-1.03 (-4.71 to -0.43)*	
	Q4 (2021)-Q3 (2022)	5.74 (0.81-8.78)*	
	Q3 (2022)-Q3 (2023)	-7.65 (-13.94 to -4.61)*	
Midwest	Q2 (2015)-Q3 (2023)	0.83 (0.17-1.59)*	0.83 (0.17-1.59)*

QPC: quarterly percent change; 95%CI: 95% confidence interval; AQPC: average quarterly percent change; *statistically significant trend.

We observed the formation of four distinct clusters, hereinafter referred to as: cluster A (n=8; 29.63%) - states of Acre, Bahia, Pará, Paraíba, Pernambuco, Rio Grande do Norte, Sergipe and Tocantins; cluster B (n=5; 18.52%) - states of Alagoas, Amazonas, Ceará, Maranhão and Piauí; cluster C (n=12; 44.44%) - Federal District and states of Espírito Santo, Goiás, Mato Grosso, Mato Grosso do Sul, Minas Gerais, Paraná, Rio de Janeiro, Rio Grande do Sul, Rondônia, Santa Catarina and São Paulo; and cluster D (n=2; 7.41%) - states of Amapá and Roraima (Table 3).

**Table 3.** Descriptive and comparative measures of state clusters for the interruption of tuberculosis treatment among people experiencing homelessness, according to socioeconomic and programmatic indicators, Brazil, 2015-2023.

Indicator	A	B	C	D	p-value*
	Average (SD)	Average (SD)	Average (SD)	Average (SD)	
Treatment interruption rate	25.41 (8.13)^a^	35.07 (4.48)^b^	33.00 (6.63)^b^	37.18 (5.44)^ab^	0.057
Human Development Index	0.71 (0.02)^a^	0.70 (0.02)^a^	0.76 (0.03)^b^	0.69 (0.01)^a^	<0.001
Gini Index	0.55 (0.03)^a^	0.53 (0.01)^ab^	0.49 (0.04)^b^	0.56 (0.05)^ab^	0.016
Percentage of those vulnerable to poverty	44.48 (5.24)^a^	48.91 (5.33)^a^	19.81 (5.84)^b^	46.40 (1.03)^a^	<0.001
Percentage of poor people	22.49 (4.33)^a^	26.04 (3.75)^a^	7.52 (2.56)^b^	26.01 (3.42)^a^	<0.001
Percentage of people in extreme poverty	11.28 (2.76)^a^	12.60 (2.43)^a^	3.79 (1.54)^b^	7.54 (0.37)^a^	<0.001
Final synthetic indicator	8.42 (0.71)^a^	9.91 (0.71)^b^	8.08 (0.68)^a^	7.01 (0.33)^ab^	0.002
AIDS incidence rate	10.10 (3.28)^a^	11.98 (2.12)^abc^	14.72 (4.70)^b^	32.43 (14.48)^c^	0.017
Percentage of hospitalizations due to primary healthcare conditions	20.79 (2.70)	25.05 (5.39)	21.09 (3.46)	21.47 (0.27)	0.313
Illiteracy rate	9.76 (3.13)^a^	11.41 (3.89)^a^	3.76 (1.70)^b^	4.87 (0.93)^ab^	0.002
Per capita income	538.19 (124.97)^a^	422.38 (53.19)^b^	797.42 (231.65)^c^	500.41 (69.49)^abc^	0.003

SD: standard deviation; AIDS: acquired immunodeficiency syndrome; *Kruskal-Wallis test; Note: different letters on the same line indicate statistically significant difference between groups (values) according to the Mann-Whitney test (p≤0.050).

There were statistically significant differences between the clusters for almost all indicators. Cluster A had the lowest average rate of treatment interruption and the lowest incidence of AIDS and rates of hospitalization from primary healthcare conditions. Cluster B had the second highest average of interruption and presented a high percentage of people in poverty and/or with low level of education and income; however, it had the best performance of primary health care (Table 3).

Cluster C, with the second lowest rate of treatment interruption, had the best rate of development, the lowest percentage of people in poverty, the highest level of education and income, and also the lowest values of inequality in income distribution. Finally, cluster D, with the highest average rate of treatment interruption, presented the lowest human development, the highest income inequality, the highest incidence of AIDS, and the worst performance of primary health care (Table 3).

## DISCUSSION

We observed the influence of different factors on the interruption of tuberculosis treatment among PEH in Brazil. In the trend analysis, characteristics - such as men, older age, non-access to social benefits, non-performance of DOT, and tobacco and drug use - significantly increased for this outcome. According to the cluster analysis, there were different groups, highlighting that income inequality and incidence of AIDS stood out in states with greater treatment interruption.

Experiencing homelessness exacerbates the chances of interruption of tuberculosis treatment, given the extreme fragility of the public in relation to health care. Under these conditions, access to information and health is scarce and insufficient, and remaining in a specific and adequate space becomes a daily challenge in the face of the everyday need to search for food, work, and place for rest, aspects that make this population extremely vulnerable, especially in the post-COVID-19 pandemic context^16^.

The national and international literature has also established profiles of greater susceptibility to discontinuity of treatment, among them, the use of chemical substances and the non-performance of DOT^5^ ^,^ ^6^ ^,^ ^17^ ^,^ ^18^. In the present study, we identified the persistence of the increasing trend of treatment interruption in PEH in light of several characteristics, which may indicate the fragility in the approach of these contexts determinants of health in current policies and strategies, especially those of social protection.

The joint work of various sectors, in addition to social assistance, is indispensable to contemplate the needs of these individuals. Actions that ensure safety, shelter provision, food, safety and treatment actions for comorbidities, including substance use, are crucial to promote adherence to tuberculosis treatment^19^. Although social protection actions are recommended^20^ ^,^ ^21^, it is assumed that, in practice, they do not adequately and universally achieve PEH.

Conversely, being a beneficiary from social programs had an increasing trend in treatment interruption, and those who did not receive it had decreasing rates. Numerous healthcare professionals have reported the positive effects of social programs on PEH, especially on the outcome of tuberculosis^22^. The fact that this variable has incompleteness greater than 40%^5^ in SINAN may have led to an unrepresentativeness of the data. Therefore, caution is recommended in the interpretation of this result.

Moreover, the absence of this datum may not be random: people who are more vulnerable or who use healthcare services less may have a greater chance of not having this variable filled in, distorting the observed relationship between receiving the benefit and interrupting treatment. In addition, if individuals who interrupt treatment are more likely to not have their complete data on SINAN, the impact of social benefit may be underestimated in the analyses.

The increasing trend of treatment interruption among people without HIV coinfection can be explained by the fact that they have a less proximal follow-up of healthcare services compared to those who have coinfection, which, overall, undergo treatment in specialized services such as outpatient clinics and hospitals. This more systematic follow-up can contribute to a more effective attachment to the treatment of tuberculosis, improving its outcomes.

However, it should be noted that HIV testing among PEH with tuberculosis is insufficient in the country, being less than 30% in some states^8^, even in view of the recommendation. This scenario raises questions about the real epidemiological scenario of tuberculosis-HIV coinfection among PEH in Brazil^20^. Thus, the different hypotheses regarding the increase or decrease of the trend must be interpreted in light of structural limitations of the country such as the lack of specific data from PEH.

Soeiro et al.^23^ showed a decreasing trend in the interruption of tuberculosis treatment in the general population in the North, Northeast, and South regions of Brazil. Nonetheless, these findings contrast with what was observed in PEH, considering that the North and Midwest regions had an increasing trend of this indicator, indicating a group that requires greater focus to achieve the national targets and the SDGs, which seek to end the tuberculosis epidemic by 2030^24^ ^,^ ^25^.

Although all states have a high occurrence of treatment interruption among PEH, because it is a notoriously vulnerable group, weaknesses in the territory can exacerbate the social determination of the disease. We observed the relationship between states with higher social and programmatic inequality and the occurrence of high treatment interruption (clusters B and D), while states with better development and income presented lower percentage (clusters A and C).

This can be partly explained by the additional challenges these locations face to effectively and equanimously implement programs and social protection strategies, as well as to operationalize and expand the care network, especially primary health care. Consequently, this leads to obstacles in the guarantee of longitudinal care to PEH with tuberculosis^26^. Observing the geoprogrammatic aspects related to the worst outcomes of the disease is indispensable for its control^8^ ^,^ ^26^.

According to the cluster analysis, there were regional inequalities that directly impact the outcomes of tuberculosis treatment, highlighting the need for care strategies adapted to localities. Disregarding these inequalities can perpetuate cycles of vulnerability and limit the effectiveness of interventions. The adoption of equitable public policies that consider factors, such as poverty and education, is essential to mitigate structural barriers and promote adherence to treatment.

The adversities and particularities that compose the care of PEH go beyond health, because they need to look closely at the lack of access to public policies as well as poverty, prejudice, exclusion, and social stigma. Thus, having professionals willing to create bonds and, in addition, producing narratives that overcome these practical obstacles is essential to reduce the interruption of tuberculosis treatment among PEH^27^, a fact that can be an additional challenge in the management of tuberculosis.

The experience of Alecrim et al.^27^ demonstrates the positive impact of the Street Clinic (*Consultório na Rua* - CnR) by promoting the development of bonds, early diagnosis, and treatment of tuberculosis. Through itinerant actions integrated with the healthcare network, the CnR teams contributed to adherence to treatment, cure of diseases, and social reintegration. The strengthening of this model can be decisive for addressing health challenges and implementing inclusive public policies​ for PEH^27^.

Another promising strategy is the Healthy Brazil Program (*Programa Brasil Saudável*), proposed in 2024, under the coordination of the Interministerial Committee for the Elimination of Tuberculosis and Other Socially Determined Diseases^28^. Its focus is to promote the collaboration of different ministries (such as education, health, and social assistance), aiming at improving access to services and reducing inequalities in the country^28^. One of the main objectives is to eliminate tuberculosis as a public health issue by 2030.

Integrated strategies, involving multiple sectors and actors, can be an effective approach to accelerating the elimination of tuberculosis. However, its implementation must be accompanied by economic and social policies that systematically address the causes and consequences of inequalities in Brazil. These injustices are critical determinants that increase and sustain vulnerability to tuberculosis, especially among PEH, resulting in the deterioration of access to fundamental rights.

It is worth mentioning that the interpretation of our findings should be considered in light of weaknesses inherent in the use of secondary sources, such as missing, incorrect, or underreported data. This may compromise the quality of the analyses and associations observed in the space conglomerate. Furthermore, the high number of municipalities with absence of cases suggests that the choice of FUs as an analysis unit may introduce a bias to inferences, given the greater aggregation of data.

In short, we identified that the temporal trend for the indicator of tuberculosis treatment interruption among homeless people is especially influenced by the following variables: sex, age, DOT performance, tobacco use, tuberculosis-HIV coinfection, and notification region. In addition, we observed the formation of four clusters, in which states with the worst social indicators had a higher percentage of discontinuity of treatment for tuberculosis in this population.

The evidence points to the need to consider the influence of social, epidemiological, programmatic, and territorial characteristics in the management of policies, programs, and healthcare services to people experiencing homelessness who have the disease. Furthermore, it is necessary to develop more research that evaluates the access of this population to healthcare services and actions, in order to understand the obstacles faced by PEH during the follow-up of antituberculosis therapy.
