# Supplementary material for: Trends and clusters of tuberculosis treatment interruption among people experiencing homelessness in Brazil: influence of individual, social and programmatic factors
Source: Rev Bras Epidemiol. 2025 Aug 8;28:e250041. doi: 10.1590/1980-549720250041 (PMC12333893; doi:10.1590/1980-549720250041)
Supplement: Translation

# Translation: Tendências e clusters da interrupção do tratamento da tuberculose nas pessoas em situação de rua no Brasil: influência de fatores individuais, sociais e programáticos

**DOI:** 10.1590/1980-549720250041.2

**Authors:** Gabriel Pavinati, Lucas Vinícius de Lima, Melisane Regina Lima Ferreira, Simone Teresinha Protti Zanatta, Gabriela Tavares Magnabosco

**Affiliations:** I Universidade Estadual de Maringá, Programa de Pós-Graduação em Enfermagem - Maringá (PR), Brasil.; II Secretaria Municipal de Saúde, Gerência de Planejamento - Maringá (PR), Brasil.; III Ministério da Saúde, Coordenação-Geral de Vigilância da Tuberculose, Micoses Endêmicas e Micobactérias Não Tuberculosas - Brasília (DF), Brasil.; IV Universidade Federal de São Carlos, Programa de Pós-Graduação em Enfermagem - São Carlos (SP), Brasil.

**Keywords:** Tuberculose, Pessoas mal alojadas, Cooperação e adesão ao tratamento, Disparidades nos níveis de saúde, Estudos ecológicos

## Abstract

**Objetivo:**

Analisar as tendências temporais e os agrupamentos estaduais dos indicadores de interrupção do tratamento da tuberculose na população em situação de rua no Brasil.

**Métodos:**

Estudo ecológico da interrupção do tratamento da tuberculose na população em situação de rua, de 2015 a 2023. Realizaram-se análises de tendência por regressão *joinpoint*, estratificadas por variáveis sociodemográficas e epidemiológicas. Aplicou-se análise pelo método *k-means* para identificar agrupamentos estaduais do desfecho, segundo indicadores socioeconômicos e programáticos.

**Resultados:**

Houve aumento da interrupção do tratamento para: sexo masculino (variação percentual trimestral média - VPTM=0,15; intervalo de confiança de 95% - IC95% 0,04-0,29), 40-59 anos (VPTM=0,38; IC95% 0,25-0,53), uso de tabaco (VPTM=0,72; IC95% 0,61-0,82), recebimento de benefícios sociais (VPTM=4,59; IC95% 3,69-6,02), ausência de tratamento diretamente observado (VPTM=0,49; IC95% 0,39-0,63), ausência de coinfecção com HIV (VPTM=0,38; IC95% 0,30-0,51), região Norte (VPTM=1,51; IC95% 0,96-2,21) e região Centro-Oeste (VPTM=0,83; IC95% 0,17-1,59). Pela análise de agrupamento, o grupo A apresentou a menor taxa de interrupção, baixa incidência de aids e melhores indicadores programáticos. O grupo B teve elevada pobreza e baixa escolaridade e renda, mas bom desempenho da atenção primária. O grupo C se destacou pelo maior desenvolvimento humano, melhores indicadores sociais e menor desigualdade. Já o grupo D concentrou os piores resultados: maior interrupção do tratamento, desigualdade, incidência de aids e fragilidade na atenção primária.

**Conclusão:**

Notaram-se desigualdades socioeconômicas e programáticas no acesso e na vinculação ao tratamento da tuberculose entre as pessoas em situação de rua no Brasil.

## INTRODUÇÃO

A estreita relação entre os aspectos socioeconômicos e a epidemia global de tuberculose sublinha a importância de incorporar os Objetivos do Desenvolvimento Sustentável (ODS) no enfrentamento da doença^1^. A Organização Mundial da Saúde (OMS) destaca que sete desses objetivos estão diretamente ligados à prevalência da tuberculose, além do objetivo três, que visa à sua eliminação ao abranger áreas como redução de desigualdades, pobreza e fome, entre outros temas^1^.

A investigação da ocorrência da tuberculose, agravo de forte determinação social, na população em situação de rua (PSR) tem sido objeto de diversos estudos. A maior carga da tuberculose na PSR foi reportada em pesquisas conduzidas em diferentes países, dentre eles: Coreia^2^, Etiópia^3^, Índia^4^, Estados Unidos da América e Brasil^5^ ^,^ ^6^. Entende-se que a extrema fragilidade social enfrentada por esse público invisibilizado é determinante na manutenção dessa problemática em saúde pública.

Globalmente, estima-se que um total global de 10,8 milhões de pessoas adoeceram por tuberculose em 2023, o equivalente a 134 casos novos a cada 100.000 habitantes^1^. No Brasil, somente em 2024, foram oficialmente registrados 84.308 casos novos de tuberculose, representando uma taxa de incidência de 39 casos a cada 100.000 habitantes^7^. Desse montante, estima-se que 3,6% foram casos entre as pessoas em situação de rua, sendo o segundo grupo vulnerável mais afetado pela doença^7^.

Metas ambiciosas foram propostas, nacional e internacionalmente, para eliminar a tuberculose como problema de saúde pública até 2035^1^ ^,^ ^2^ ^,^ ^3^ ^,^ ^4^ ^,^ ^5^ ^,^ ^6^ ^,^ ^7^. No entanto, persistem entraves no acesso e na vinculação das pessoas afetadas aos serviços de saúde, ocasionando um fenômeno relevante, sobretudo na PSR: a interrupção do tratamento^6^ ^,^ ^8^ ^,^ ^9^. No Brasil, estudo evidenciou que a cura da tuberculose entre as pessoas em situação de rua pode ser inferior a 50% em alguns estados, ao passo que a interrupção pode superar os 40%^8^.

Diferentes fatores podem explicar a maior suscetibilidade da PSR à interrupção do tratamento, tais como: diminuição da idade, forma pulmonar da tuberculose, coinfecção pelo HIV e uso de drogas^5^. Eles podem, todavia, ser minimizados com intervenções como o tratamento diretamente observado (TDO) e os auxílios financeiros^5^. Apesar disso, o comportamento temporal dos indicadores de cura da tuberculose no Brasil é preocupante: tendências decrescentes foram reportadas na maioria dos estados^10^.

A tuberculose, como um marcador de desigualdades sociais, apresenta desafios adicionais na PSR. Além das barreiras estruturais enfrentadas por essa população, como a falta de moradia e a insegurança alimentar, questões relacionadas à estigmatização e à discriminação também contribuem para a exclusão dos serviços de saúde. Isso tudo, afinal, intensifica as dificuldades de implementação efetiva do tratamento, exigindo intervenções intersetoriais com enfoque na redução das iniquidades.

Ao compreender o comportamento temporal da interrupção do tratamento da tuberculose na PSR, considerando potenciais fatores com influência na adesão e na vinculação, e as áreas mais vulneráveis à sua ocorrência, mediante aspectos socioeconômicos e programáticos, este estudo pode ser crucial para orientar estratégias balizadas pelos ODS. Portanto, analisaram-se as tendências temporais e os agrupamentos estaduais dos indicadores de interrupção do tratamento da tuberculose na PSR no Brasil.

## MÉTODOS

Estudo ecológico reportado com base no *Reporting of Studies Conducted using Observational Routinely-Collected Health Data*. Teve-se como foco a avaliação dos indicadores de interrupção do tratamento para tuberculose na PSR, mediante análises de séries temporais e de clusterização não hierárquica. Considerou-se como cenário do estudo o Brasil, que é dividido político-administrativamente em 27 Unidades Federativas (UFs), contempladas por 450 regiões de saúde e 5.570 municípios e distritos.

A PSR é definida como um grupo populacional heterogêneo, que possui em comum a pobreza extrema, os vínculos familiares interrompidos ou fragilizados, e a inexistência de moradia convencional regular^11^. Dados do Ministério dos Direitos Humanos e da Cidadania indicam que, em 2022, havia 236.400 pessoas em situação de rua, com a devida inscrição no Cadastro Único para Programas Sociais (Cadastro Único), ou seja, 1 em cada 1.000 pessoas no país estava vivendo em situação de rua^11^.

Para este estudo, os dados foram extraídos do Sistema de Informação de Agravos de Notificação (Sinan) em 4 de agosto de 2024, na seção “Transferência de Arquivos”, e tabulados pelo TabWin (disponível em: https://datasus.saude.gov.br/transferencia-de-arquivos/). Foram desconsiderados todos os casos em que as variáveis de interesse estivessem em branco ou preenchidas como “ignorado”, assumindo que essas informações não possibilitariam a análise adequada e fidedigna dos casos notificados.

Incluíram-se os casos com a variável “pessoa em situação de rua” preenchida como “sim”. Para o recorte temporal, determinou-se o período de 2015 a 2023, dado que a inserção das variáveis para populações especiais (i.e., PSR) na ficha de notificação ocorreu em 2015. Como critério de inclusão, postulou-se: pessoas adultas, consideradas aquelas com ≥18 anos, que tivessem iniciado o tratamento da tuberculose como caso novo e que tivessem o encerrado como interrupção do tratamento (i.e., abandono).

Como caso novo compreenderam-se aqueles com os tipos de entrada “caso novo” e “não sabe”, semelhante à definição estabelecida pelo Ministério da Saúde^11^. Como interrupção do tratamento foram considerados todos os casos de tuberculose que tiveram como situação de encerramento “abandono” ou “abandono primário”. Para delimitação do ano de registro, considerou-se a variável “ano de diagnóstico”; e, para o referenciamento do local de atendimento, utilizou-se a variável “UF de notificação”.

A construção do indicador de interrupção do tratamento para tuberculose na PSR se baseou no relatório do “Caderno de Indicadores da Tuberculose”^12^, do Ministério da Saúde do Brasil. O cálculo se deu da seguinte maneira: divisão do número de casos novos com desfecho de interrupção do tratamento em determinado período e população (numerador) pelo número total de casos novos registrados, no mesmo período e com a mesma população (denominador), e o resultado multiplicado por 100.

Para as análises de tendência, empregou-se o método *joinpoint*, permitindo a localização de pontos de inflexão nas séries^13^. As análises foram estratificadas por: região de notificação (Norte; Nordeste; Sudeste; Sul; Centro-Oeste); sexo (masculino; feminino); faixa etária (em anos: 18-39; 40-59; ≥60 ou mais); beneficiário de programas sociais (sim; não); HIV (positivo; negativo); uso de drogas (sim; não), tabaco (sim; não), ou álcool (sim; não); diabetes (sim; não); e realização do TDO (sim; não).

Para a análise das características dos indivíduos que interromperam o tratamento, optou-se por utilizar um denominador comum, correspondente ao total de interrupções registradas em cada ano. Essa abordagem visou identificar mudanças na composição desse desfecho ao longo do tempo, permitindo observar quais subgrupos passaram a ser mais representativos. Não se trata de estimativa de risco, mas de uma estratégia descritiva voltada à compreensão do perfil predominante entre os casos de interrupção.

Como a análise de séries temporais pressupõe um número mínimo de oito pontos para que as tendências sejam estimadas de forma adequada^13^, optou-se por utilizar indicadores trimestrais, totalizando 36 períodos. Entretanto, como um número reduzido de eventos poderia gerar variações aleatórias importantes nas medições de porcentagem^12^, empregou-se a estratégia de suavização da série por média móvel de três pontos, o que resultou em séries com 34 pontos (considerados como os trimestres suavizados).

Os indicadores trimestrais de interrupção do tratamento para tuberculose foram transformados por função logarítmica natural devido à melhor interpretação e comparação dos resultados. Os modelos log-lineares foram ajustados pelos erros-padrão dos percentuais e pela correção da autocorrelação de primeira ordem, quando verificada a partir dos dados^13^. Os modelos finais, estimados via pesquisa em grade, foram escolhidos pelo menor valor do critério de informação bayesiano ponderado^13^.

Para cada modelo final, foram calculadas as variações percentuais trimestrais (VPT) referentes à mudança dos indicadores em cada *joinpoint*, as variações percentuais trimestrais médias (VPTM) relativas às médias geométricas das VPT, e os intervalos de confiança de 95% (IC95%). Na interpretação, VPT/VPTM positivas indicaram tendência crescente, enquanto as VPT/VPTM negativas, tendência de declínio. Foram consideradas significativas as VPT/VPTM cujos IC95% não incluíssem o valor nulo.

Para mais, procedeu-se à análise de agrupamento não hierárquico pelo método *k-means*, que divide o conjunto de dados em *k* grupos, reduzindo a soma das distâncias quadradas entre os pontos e seus respectivos centroides. Toma-se como pressuposto que os dados dentro de cada grupo (*k*) sejam mais semelhantes entre si do que com os dados de outros grupos, tendo-se como parâmetro a distância entre os pontos e os centroides. Para este estudo, o valor de *k* foi definido pelo método *elbow* (cotovelo).

Como unidades de análise, foram definidos os 26 estados brasileiros e o Distrito Federal. As variáveis consideradas mediadoras no agrupamento foram selecionadas de acordo com a proximidade teórica com o desfecho em estudo - interrupção do tratamento para tuberculose -, conforme estudos prévios^5^ ^,^ ^14^ ^,^ ^15^. A descrição detalhada de cada indicador está apresentada no Quadro 1. As variáveis de cada grupo foram descritas em função da média aritmética e do desvio padrão (DP).

**Quadro 1.** Indicadores estaduais provenientes do Instituto Brasileiro de Geografia e Estatística (IBGE), do Departamento de Informação e Informática do Sistema Único de Saúde (Datasus) e do Sistema de Informação em Saúde para a Atenção Básica (Sisab).

Indicador	Descrição
Índice de desenvolvimento humano	Avalia o progresso a longo prazo em três dimensões fundamentais: vida longa e saudável, acesso ao conhecimento e padrão de vida digno. Os indicadores finais são consolidados em uma escala que varia de 0,00 a 1,00, onde um valor mais próximo de 1,00 indica um desenvolvimento geral melhor.
Índice de Gini	Mede o grau de desigualdade na distribuição da renda domiciliar *per capita*. O valor do índice varia de 0,00 a 1,00, onde 0,00 representa igualdade perfeita (todos têm a mesma renda) e 1,00 representa desigualdade perfeita (uma pessoa tem toda a renda enquanto as outras não têm nada).
Percentual de vulneráveis à pobreza	Número de indivíduos com renda domiciliar *per capita* igual ou inferior a 255,00 reais por mês, dividido pela população total; o resultado foi multiplicado por 100.
Percentual de pobres	Número de indivíduos com renda domiciliar *per capita* igual ou inferior a 140,00 reais por mês, dividido pela população total; o resultado foi multiplicado por 100.
Percentual de extremamente pobres	Número de indivíduos com renda domiciliar *per capita* igual ou inferior a 70,00 reais por mês, dividido pela população total; o resultado foi multiplicado por 100.
Indicador sintético final	O indicador sintético final do Programa Previne Brasil é o cálculo final de um conjunto de sete indicadores de desempenho da atenção primária. Seu valor varia de zero a dez, sendo obtido a partir da atribuição da nota individual para cada indicador e da ponderação por seus respectivos pesos.
Taxa de incidência de aids	Número de casos recém-diagnosticados de síndrome da imunodeficiência adquirida (códigos B20 a B24 da Classificação Internacional de Doenças - 10ª edição) registrados no ano considerado, dividido pela população total; o resultado foi multiplicado por 100.000.
Percentual de internações por condições sensíveis à atenção primária	Percentual de internações hospitalares por condições sensíveis à atenção primária (conforme lista da Classificação Internacional de Doenças - 10ª edição), em relação ao número total de internações clínicas, em determinado espaço geográfico e ano.
Taxa de analfabetismo	População com 18 anos ou mais que não consegue ler ou escrever uma nota simples, dividida pelo número total de pessoas nessa faixa etária; o resultado foi multiplicado por 100.
Renda *per capita*	Soma da renda (em reais) de todos os indivíduos residentes em domicílios particulares permanentes, dividida pelo número total desses indivíduos.

Os grupos obtidos pela análise de agrupamento *k-means* foram comparados estatisticamente por meio do teste de Kruskal-Wallis, que verifica se há diferenças significativas na distribuição de uma variável entre três ou mais grupos independentes a partir dos postos (*ranks*) dos valores. Na presença de significância, isto é, p≤0,050, aplicou-se o teste de Mann-Whitney para comparações par a par, avaliando se as distribuições de dois grupos diferem entre si, sem pressupor normalidade dos dados.

Para as análises, utilizaram-se o *Joinpoint Regression Program* ^*®*^ (versão 5.0.2) e o SPSS Statistics^®^ (versão 25.0). Pontua-se que este estudo integra a tese de doutorado “Desafios na continuidade do tratamento da tuberculose na população em situação de rua: proteção social e padrões ecológicos no contexto brasileiro”, apresentada ao Programa de Pós-Graduação em Enfermagem da Universidade Estadual de Maringá. Obteve-se aprovação do Comitê de Ética em Pesquisa (parecer nº 6.736.365/2024).

### Declaração de disponibilidade de dados

Todo o conjunto de dados que dá suporte aos resultados deste estudo foi disponibilizado no Mendeley Data e pode ser acessado em https://doi.org/10.17632/zfypkvyhg3.1.

## RESULTADOS

De 2015 a 2023 foram registrados 18.474 casos de tuberculose na PSR no Brasil, com variação nos desfechos do tratamento ao longo dos anos (Figura 1). Desse montante, 5.981 (32,38%) evoluíram com a cura da doença, 2.747 (14,87%) foram a óbito, 1.920 (10,39%) tiveram outros desfechos (como falência, transferência, etc.), 1.188 (6,43%) não tiveram registro de encerramento do tratamento e 6.638 (35,93%) interromperam o tratamento da tuberculose - sendo este o universo de casos do presente estudo.

**Figura 1.** Percentuais anuais dos desfechos do tratamento da tuberculose na população em situação de rua, segundo cura, interrupção do tratamento, óbito, ignorado e outro desfecho, Brasil, 2015-2023.

No tocante às características, houve predominância de interrupção do tratamento entre pessoas do sexo masculino, de 18 a 39 anos, que faziam uso de álcool, tabaco e drogas ilícitas e que se localizavam na região Sudeste. Ao longo dos anos, prevaleceu a ausência de realização do TDO e o recebimento de benefícios de programas sociais. A maioria não apresentou diabetes ou HIV; no entanto, destaca-se que a ocorrência da coinfecção tuberculose-HIV superou 20% dos casos em todos os anos (Tabela 1).

**Tabela 1.** Percentuais anuais de interrupção do tratamento da tuberculose na população em situação de rua, segundo características demográficas, socioeconômicas e epidemiológicas, Brasil, 2015-2023.

Variável	Período								
	2015	2016	2017	2018	2019	2020	2021	2022	2023
Sexo									
Masculino	79,15	80,28	81,67	83,33	81,72	83,24	81,72	82,66	83,42
Feminino	20,85	19,72	18,33	16,67	18,28	16,76	18,28	17,34	16,58
Faixa etária (anos)									
18-39	59,22	58,98	60,28	59,64	56,54	55,80	52,69	55,15	55,25
40-59	36,50	37,73	35,86	36,71	39,20	40,68	43,71	40,53	40,55
≥60	4,28	3,29	3,86	3,65	4,26	3,52	3,60	4,32	4,20
Tratamento diretamente observado									
Sim	35,37	32,78	31,40	29,25	30,76	26,29	25,11	27,70	25,49
Não	64,63	67,22	68,60	70,75	69,24	73,71	74,89	72,30	74,51
Diabetes									
Sim	3,35	2,81	3,97	3,21	3,03	3,15	2,89	2,99	3,77
Não	96,65	97,19	96,03	96,79	96,97	96,85	97,11	97,01	96,23
Uso de álcool									
Sim	52,31	55,34	53,27	52,96	52,26	54,85	56,25	57,05	54,53
Não	47,69	44,66	46,73	47,04	47,74	45,15	43,75	42,95	45,47
Uso de tabaco									
Sim	45,86	46,64	49,85	49,59	52,42	53,68	51,60	56,81	57,31
Não	54,14	53,36	50,15	50,41	47,58	46,32	48,40	43,19	42,69
Uso de drogas									
Sim	57,91	59,92	62,19	64,19	62,35	63,77	66,16	65,64	65,28
Não	42,09	40,08	37,81	35,81	37,65	36,23	33,84	34,36	34,72
Beneficiário de programas sociais									
Sim	4,43	7,12	8,13	9,85	8,59	10,37	9,81	11,92	15,35
Não	95,57	92,88	91,87	90,15	91,41	89,63	90,19	88,08	84,65
Coinfecção com HIV									
Sim	28,39	26,37	29,83	30,68	25,98	23,84	23,10	22,61	21,48
Não	71,61	73,63	70,17	69,32	74,02	76,16	76,90	77,39	78,52
Região de notificação									
Norte	3,96	4,63	7,05	6,11	9,88	7,74	8,71	9,86	7,72
Nordeste	11,71	13,46	15,60	11,96	12,25	11,38	11,35	11,61	9,98
Sudeste	60,45	63,12	58,74	59,63	57,54	60,67	58,96	57,02	62,25
Sul	19,62	14,88	14,48	16,76	16,48	15,10	15,58	16,81	14,04
Centro-Oeste	4,26	3,91	4,13	5,54	3,85	5,11	5,40	4,70	6,01

Houve tendência crescente da interrupção do tratamento da tuberculose para: sexo masculino (VPTM=0,15; IC95% 0,04-0,29), 40-59 anos (VPTM=0,38; IC95% 0,25-0,53), ausência de TDO (VPTM=0,49; IC95% 0,39-0,63), uso de tabaco (VPTM=0,72; IC95% 0,61-0,82), recebimento de benefícios sociais (VPTM=4,59; IC95% 3,69-6,02), ausência de coinfecção com HIV (VPTM=0,38; IC95% 0,30-0,51) e regiões Norte (VPTM=1,51; IC95% 0,96-2,21) e Centro-Oeste (VPTM=0,83; IC95% 0,17-1,59), conforme Tabela 2.

**Tabela 2.** Tendência temporal dos percentuais de interrupção do tratamento da tuberculose na população em situação de rua, segundo características demográficas, socioeconômicas e epidemiológicas, Brasil, 2015-2023.

Variável	Trimestre (Ano)	VPT (IC95%)	VPTM (IC95%)
Sexo			
Masculino	T2 (2015)-T2 (2018)	0,39 (0,14-2,39)*	0,15 (0,04-0,29)*
	T2 (2018)-T3 (2023)	0,01 (-0,87-0,11)	
Feminino	T2 (2015)-T4 (2018)	-1,97 (-2,78 a -1,41)*	-0,62 (-0,90 a -0,36)*
	T4 (2018)-T4 (2019)	4,15 (0,83-7,79)*	
	T4 (2019)-T3 (2020)	-5,87 (-8,68 a -1,66)*	
	T3 (2020)-T1 (2022)	3,62 (2,40-8,88)*	
	T1 (2022)-T4 (2022)	-8,73 (-11,39 a -4,78)*	
	T4 (2022)-T3 (2023)	5,25 (1,40-13,49)*	
Faixa etária (anos)			
18-39	T2 (2015)-T1 (2018)	0,18 (-0,19-0,88)	-0,15 (-0,26 a -0,02)*
	T1 (2018)-T3 (2021)	-0,90 (-2,53 a -0,63)*	
	T3 (2021)-T3 (2023)	0,70 (0,09-2,00)*	
40-59	T2 (2015)-T3 (2016)	1,31 (0,38-3,38)*	0,38 (0,25-0,53)*
	T3 (2016)-T3 (2017)	-2,31 (-4,04 a -0,77)*	
	T3 (2017)-T3 (2021)	1,45 (1,17-1,79)*	
	T3 (2021)-T3 (2022)	-2,46 (-4,03-1,36)	
	T3 (2022)-T3 (2023)	0,57 (-1,00-3,23)	
≥60	T2 (2015)-T3 (2023)	0,41 (-0,08-0,95)	0,41 (-0,08-0,95)
Tratamento diretamente observado			
Sim	T2 (2015)-T3 (2023)	-0,94 (-1,23 a -0,66)*	-0,94 (-1,23 a -0,66)*
Não	T2 (2015)-T2 (2018)	1,05 (0,73-1,56)*	0,49 (0,39-0,63)*
	T2 (2018)-T1 (2019)	-2,89 (-3,96 a -0,37)*	
	T1 (2019)-T4 (2020)	2,14 (1,36-4,26)*	
	T4 (2020)-T2 (2022)	-1,17 (-2,93 a -0,29)*	
	T2 (2022)-T3 (2023)	0,96 (0,04-0,63)*	
Diabetes			
Sim	T2 (2015)-T3 (2023)	-0,01 (-1,04-1,19)	-0,01 (-1,04-1,19)
Não	T2 (2015)-T3 (2023)	0,00 (-0,04-0,04)	0,00 (-0,04-0,04)
Uso de álcool			
Sim	T2 (2015)-T4 (2016)	1,33 (0,46-4,03)*	0,05 (-0,14-0,26)
	T4 (2016)-T4 (2017)	-2,18 (-3,89 a -0,41)*	
	T4 (2017)-T4 (2022)	0,57 (0,43-0,93)*	
	T4 (2022)-T3 (2023)	-2,96 (-7,03 a -0,72)*	
Não	T2 (2015)-T4 (2016)	-1,58 (-4,75 a -0,39)*	-0,06 (-0,29-0,14)
	T4 (2016)-T4 (2017)	2,71 (0,66-5,10)*	
	T4 (2017)-T4 (2022)	-0,69 (-1,23 a -0,51)*	
	T4 (2022)-T3 (2023)	3,63 (0,54-8,11)*	
Uso de tabaco			
Sim	T2 (2015)-T2 (2020)	0,92 (0,76-1,13)*	0,72 (0,61-0,82)*
	T2 (2020)-T3 (2021)	-1,36 (-3,96 a -0,33)*	
	T3 (2021)-T2 (2022)	4,28 (2,19-5,68)*	
	T2 (2022)-T3 (2023)	-0,05 (-1,76-0,68)	
Não	T2 (2015)-T2 (2020)	-0,90 (-1,12 a -0,73)*	-0,77 (-0,89 a -0,64)*
	T2 (2020)-T3 (2021)	1,40 (-0,07-3,84)	
	T3 (2021)-T2 (2022)	-4,78 (-6,16 a -2,27)*	
	T2 (2022)-T3 (2023)	0,03 (-1,20-2,86)	
Uso de drogas			
Sim	T2 (2015)-T3 (2018)	0,97 (0,80-1,24)*	0,37 (0,31-0,45)*
	T3 (2018)-T2 (2019)	-1,87 (-2,55 a -0,39)*	
	T2 (2019)-T3 (2021)	0,93 (0,65-2,12)*	
	T3 (2021)-T3 (2023)	-0,36 (-0,81 a -0,05)*	
Não	T2 (2015)-T3 (2018)	-1,42 (-1,94 a -1,09)*	-0,58 (-0,70 a -0,45)*
	T3 (2018)-T4 (2019)	1,59 (0,27-4,00)*	
	T4 (2019)-T3 (2021)	-2,10 (-4,92 a -1,30)*	
	T3 (2021)-T3 (2023)	0,79 (0,13-2,15)*	
Beneficiário de programas sociais			
Sim	T2 (2015)-T4 (2016)	15,02 (6,84-34,50)*	4,59 (3,69-6,02)*
	T4 (2016)-T4 (2021)	0,69 (-2,51-1,63)	
	T4 (2021)-T3 (2023)	7,48 (3,93-15,39)*	
Não	T2 (2015)-T4 (2016)	-0,90 (-1,90 a -0,48)*	-0,45 (-0,55 a -0,38)*
	T4 (2016)-T1 (2022)	-0,09 (-0,18-0,13)	
	T1 (2022)-T3 (2023)	-1,25 (-2,36 a -0,71)*	
Coinfecção com HIV			
Sim	T2 (2015)-T1 (2016)	-5,53 (-10,63 a -1,63)*	-1,09 (-1,34 a -0,87)*
	T1 (2016)-T2 (2018)	2,80 (1,89-4,57)*	
	T2 (2018)-T1 (2020)	-5,11 (-7,24-1,31)	
	T1 (2020)-T4 (2020)	3,02 (-5,19-4,99)	
	T4 (2020)-T3 (2023)	-1,47 (-3,67 a -0,81)*	
Não	T2 (2015)-T1 (2016)	2,58 (1,18-4,94)*	0,38 (0,30-0,51)*
	T1 (2016)-T1 (2018)	-1,35 (-2,33 a -0,93)*	
	T1 (2018)-T1 (2020)	1,72 (-0,50-2,59)	
	T1 (2020)-T4 (2020)	-0,86 (-1,39-2,27)	
	T4 (2020)-T3 (2023)	0,44 (0,16-1,19)*	
Região de notificação			
Norte	T2 (2015)-T1 (2016)	-10,20 (-24,62 a -0,26)*	1,51 (0,96-2,21)*
	T1 (2016)-T1 (2017)	23,78 (15,93-36,61)*	
	T1 (2017)-T3 (2018)	-4,35 (-9,90 a -1,49)*	
	T3 (2018)-T3 (2019)	18,62 (12,90-26,78)*	
	T3 (2019)-T3 (2020)	-11,56 (-17,09 a -6,81)*	
	T3 (2020)-T2 (2022)	6,00 (4,09-10,53)*	
	T2 (2022)-T3 (2023)	-7,11 (-11,09 a -4,35)*	
Nordeste	T2 (2015)-T2 (2017)	5,50 (3,21-10,12)*	-0,04 (-0,45-0,59)
	T2 (2017)-T1 (2018)	-9,29 (-12,42 a -1,51)*	
	T1 (2018)-T3 (2023)	-0,67 (-1,30-1,30)	
Sudeste	T2 (2015)-T2 (2022)	-0,26 (-0,96 a -0,04)*	0,08 (-0,22-0,32)
	T2 (2022)-T3 (2023)	2,01 (-0,08-6,85)	
Sul	T2 (2015)-T4 (2016)	-7,39 (-10,51 a -5,11)*	-1,47 (-1,88 a -1,12)*
	T4 (2016)-T3 (2018)	3,75 (1,59-9,67)*	
	T3 (2018)-T4 (2021)	-1,03 (-4,71 a -0,43)*	
	T4 (2021)-T3 (2022)	5,74 (0,81-8,78)*	
	T3 (2022)-T3 (2023)	-7,65 (-13,94 a -4,61)*	
Centro-Oeste	T2 (2015)-T3 (2023)	0,83 (0,17-1,59)*	0,83 (0,17-1,59)*

VPT: variação percentual trimestral; IC95%: intervalo de confiança de 95%; VPTM: variação percentual trimestral média; *tendência estatisticamente significativa.

Observou-se a formação de quatro *clusters* distintos, doravante denominados: *cluster* A (n=8; 29,63%) - Acre, Bahia, Pará, Paraíba, Pernambuco, Rio Grande do Norte, Sergipe e Tocantins; *cluster* B (n=5; 18,52%) - Alagoas, Amazonas, Ceará, Maranhão e Piauí; *cluster* C (n=12; 44,44%) - Distrito Federal, Espírito Santo, Goiás, Mato Grosso, Mato Grosso do Sul, Minas Gerais, Paraná, Rio de Janeiro, Rio Grande do Sul, Rondônia, Santa Catarina e São Paulo; e *cluster* D (n=2; 7,41%) - Amapá e Roraima (Tabela 3).

**Tabela 3.** Medidas descritivas e comparativas dos agrupamentos estaduais de interrupção do tratamento da tuberculose na população em situação de rua, segundo indicadores socioeconômicos e programáticos, Brasil, 2015-2023.

Indicador	A	B	C	D	p-valor*
	Média (DP)	Média (DP)	Média (DP)	Média (DP)	
Taxa de interrupção do tratamento	25,41 (8,13)^a^	35,07 (4,48)^b^	33,00 (6,63)^b^	37,18 (5,44)^ab^	0,057
Índice de desenvolvimento humano	0,71 (0,02)^a^	0,70 (0,02)^a^	0,76 (0,03)^b^	0,69 (0,01)^a^	<0,001
Índice de Gini	0,55 (0,03)^a^	0,53 (0,01)^ab^	0,49 (0,04)^b^	0,56 (0,05)^ab^	0,016
Percentual de vulneráveis à pobreza	44,48 (5,24)^a^	48,91 (5,33)^a^	19,81 (5,84)^b^	46,40 (1,03)^a^	<0,001
Percentual de pobres	22,49 (4,33)^a^	26,04 (3,75)^a^	7,52 (2,56)^b^	26,01 (3,42)^a^	<0,001
Percentual de extremamente pobres	11,28 (2,76)^a^	12,60 (2,43)^a^	3,79 (1,54)^b^	7,54 (0,37)^a^	<0,001
Indicador sintético final	8,42 (0,71)^a^	9,91 (0,71)^b^	8,08 (0,68)^a^	7,01 (0,33)^ab^	0,002
Taxa de incidência de AIDS	10,10 (3,28)^a^	11,98 (2,12)^abc^	14,72 (4,70)^b^	32,43 (14,48)^c^	0,017
Percentual de internações por condições da atenção primária	20,79 (2,70)	25,05 (5,39)	21,09 (3,46)	21,47 (0,27)	0,313
Taxa de analfabetismo	9,76 (3,13)^a^	11,41 (3,89)^a^	3,76 (1,70)^b^	4,87 (0,93)^ab^	0,002
Renda *per capita*	538,19 (124,97)^a^	422,38 (53,19)^b^	797,42 (231,65)^c^	500,41 (69,49)^abc^	0,003

DP: desvio padrão; AIDS: síndrome da imunodeficiência adquirida; *teste de Kruskal-Wallis; Nota: letras diferentes na mesma linha indicam diferença estatisticamente significativa entre os grupos (valores) de acordo com o teste de Mann-Whitney (p≤0,050).

Houve diferenças estatisticamente significativas entre os grupos para quase todos os indicadores. O grupo A teve a menor taxa média de interrupção do tratamento e congregou a menor incidência de aids e as menores taxas de internação por condições da atenção primária. O grupo B teve a segunda maior média de interrupção e apresentou elevado percentual de pessoas em situação de pobreza e/ou com baixa escolaridade e renda; no entanto, teve o melhor desempenho da atenção primária (Tabela 3).

O grupo C, com a segunda menor taxa de interrupção do tratamento, teve o melhor índice de desenvolvimento, os menores percentuais de pessoas em situação de pobreza, o maior nível de escolaridade e renda e, também, os menores valores de desigualdade na distribuição de renda. Por fim, o grupo D, com a maior taxa média de interrupção do tratamento, apresentou o menor desenvolvimento humano, a maior desigualdade de renda, a maior incidência de aids e o pior desempenho da atenção primária (Tabela 3).

## DISCUSSÃO

Observou-se a influência de diferentes fatores na interrupção do tratamento da tuberculose entre a PSR no Brasil. Na análise de tendência, características como sexo masculino, idade mais avançada, não acesso a benefícios sociais, não realização do TDO e uso de tabaco e drogas tiveram incrementos significativos para esse desfecho. A análise de agrupamento revelou grupos distintos, destacando que desigualdade de renda e incidência de aids se sobressaíram em estados com maior interrupção do tratamento.

A vivência nas ruas exacerba as chances de interrupção do tratamento da tuberculose, dada a extrema fragilidade do público frente aos cuidados em saúde. Nessas condições, o acesso à informação e à saúde é escasso e insuficiente, e manter-se em um espaço específico e adequado se torna um desafio diário ante a necessidade cotidiana de busca por alimentação, trabalho e local para descanso, aspectos que tornam essa população extremamente vulnerável, sobretudo no pós-pandemia da COVID-19^16^.

A literatura nacional e internacional também já estabeleceu perfis de maior suscetibilidade à descontinuidade do tratamento; dentre eles, destacam-se o uso de substâncias químicas e a não realização do TDO^5^ ^,^ ^6^ ^,^ ^17^ ^,^ ^18^. O presente estudo identificou a persistência da tendência crescente da interrupção do tratamento na PSR sob à luz de diversas características, o que pode indicar a fragilidade na abordagem desses contextos determinantes da saúde nas atuais políticas e estratégias, especialmente aquelas de proteção social.

O trabalho conjunto de diversos setores, sobremaneira com a assistência social, é indispensável para contemplar as necessidades desses indivíduos. Ações de garantia de segurança, oferta de abrigo, alimentação, segurança e tratamento para comorbidades, incluindo o uso de substâncias, são cruciais para promover a adesão ao tratamento da tuberculose^19^. A despeito das ações de proteção social serem recomendadas^20^ ^,^ ^21^, conjectura-se que, na prática, elas não alcançam adequada e universalmente a PSR.

Contraditoriamente, receber benefícios de programas sociais teve tendência crescente na interrupção do tratamento, sendo que aqueles que não o recebem tiveram taxas decrescentes. Inúmeros profissionais de saúde já reportaram os efeitos positivos dos programas sociais à PSR, sobretudo no desfecho da tuberculose^22^. O fato dessa variável ter incompletude superior a 40%^5^ no Sinan pode ter levado a uma sub-representatividade dos dados. Portanto, recomenda-se cautela na interpretação desse resultado.

Além disso, a ausência desse dado pode não ser aleatória: pessoas mais vulneráveis ou que utilizam menos os serviços de saúde podem ter maior chance de não terem essa variável preenchida, distorcendo a relação observada entre o recebimento do benefício e a interrupção do tratamento. Além disso, se indivíduos que interrompem o tratamento têm maior probabilidade de não terem seus dados completos no Sinan, o impacto do benefício social pode estar sendo subestimado nas análises.

A tendência crescente na interrupção do tratamento entre as pessoas sem coinfecção com HIV pode ser explicada pelo fato de que elas possuem um acompanhamento menos proximal dos serviços de saúde em relação àquelas que têm a coinfecção, que, de modo geral, realizam o tratamento em serviços especializados, como ambulatórios e hospitais. Esse acompanhamento mais sistemático pode colaborar para uma vinculação mais efetiva ao tratamento da tuberculose, melhorando seus desfechos.

Todavia, há que se pontuar que a testagem para HIV entre a PSR com tuberculose é insuficiente no país, sendo inferior a 30% em alguns estados^8^, mesmo diante da recomendação. Tal cenário levanta questionamentos sobre o real cenário epidemiológico da coinfecção tuberculose-HIV entre a PSR no Brasil^20^. Desse modo, as diferentes hipóteses em relação ao aumento ou à diminuição da tendência precisam ser interpretadas à luz de limitações estruturais do país, como a falta de dados específicos da PSR.

O estudo de Soeiro et al.^23^ evidenciou tendência decrescente na interrupção do tratamento da tuberculose na população geral nas regiões Norte, Nordeste e Sul do Brasil. No entanto, esses achados contrastam com o que foi observado na PSR, visto que as regiões Norte e Centro-Oeste tiveram tendência crescente desse indicador, sinalizando um grupo que precisa de maior enfoque para se alcançar as metas nacionais e os ODS, que buscam acabar com a epidemia de tuberculose até 2030^24^ ^,^ ^25^.

Apesar de todos os estados possuírem elevada ocorrência de interrupção do tratamento entre a PSR, por se tratar de um grupo notoriamente vulnerabilizado, as fragilidades no território podem exacerbar a determinação social da doença. Observou-se a relação entre os estados com maior desigualdade social e programática e a ocorrência de elevada interrupção do tratamento (*clusters* B e D), ao passo que os estados com melhor desenvolvimento e renda apresentaram menor porcentagem (*clusters* A e C).

Parte disso pode ser explicada pelos desafios adicionais que essas localidades enfrentam para implementar, de forma eficiente e equânime, programas e estratégias de proteção social, bem como para operacionalizar e expandir a rede de atenção, sobretudo a atenção primária. Consequentemente, isso gera entraves na garantia do cuidado longitudinal à PSR com tuberculose^26^. Observar os aspectos geoprogramáticos relacionados aos piores desfechos da doença é indispensável para seu controle^8^ ^,^ ^26^.

A análise de *clusters* evidenciou desigualdades regionais que impactam diretamente os desfechos do tratamento da tuberculose, destacando a necessidade de estratégias de cuidado adaptadas às localidades. Ignorar essas desigualdades pode perpetuar ciclos de vulnerabilidade e limitar a efetividade das intervenções. A adoção de políticas públicas equitativas que considerem fatores como pobreza e educação é essencial para mitigar as barreiras estruturais e promover a adesão ao tratamento.

As adversidades e particularidades que compõem o cuidado da PSR vão além da saúde, pois necessitam de olhar atento para a falta de acesso às políticas públicas, bem como para a miséria, o preconceito, a exclusão e o estigma social. Dessa forma, ter profissionais dispostos a construir vínculos e, além disso, produzir narrativas que superem esses entraves práticos é imprescindível para reduzir a interrupção do tratamento da tuberculose na PSR^27^, fato que pode ser desafio adicional no manejo da tuberculose.

A experiência de Alecrim et al.^27^ demonstra a repercussão positiva do Consultório na Rua (CnR) ao promover a criação de vínculos, o diagnóstico precoce e o tratamento da tuberculose. Por meio de ações itinerantes e integradas à rede de atenção à saúde, as equipes do CnR contribuíram para a adesão ao tratamento, a cura de doenças e a reinserção social. O fortalecimento desse modelo pode ser determinante para enfrentar os desafios de saúde e efetivar políticas públicas inclusivas​ para a PSR^27^.

Outra estratégia promissora é o Programa Brasil Saudável, proposto em 2024, sob a coordenação do Comitê Interministerial para a Eliminação da Tuberculose e Outras Doenças Socialmente Determinadas^28^. Seu foco é promover a colaboração de diferentes ministérios (como educação, saúde e assistência social), visando melhorar o acesso aos serviços e reduzir as desigualdades presentes no país^28^. Um dos principais objetivos é eliminar a tuberculose como um problema de saúde pública até 2030.

Estratégias integradas, envolvendo múltiplos setores e atores, podem ser uma abordagem eficaz para acelerar a eliminação da tuberculose. Contudo, sua implementação necessita ser acompanhada por políticas econômicas e sociais que abordem de forma sistemática as causas e as consequências das desigualdades no Brasil. Essas injustiças são determinantes críticos que aumentam e sustentam a vulnerabilidade à tuberculose, especialmente na PSR, resultando na erosão do acesso a direitos fundamentais.

Cabe mencionar que a interpretação dos achados deste estudo precisa ser considerada à luz de fragilidades inerentes ao uso de fontes secundárias, como dados faltantes, incorretos ou sub-registrados. Isso pode comprometer a qualidade das análises e das associações observadas no conglomerado espacial. Ainda, o elevado número de municípios com ausência de casos sugere que a escolha das UFs como unidade de análise pode introduzir um viés nas inferências, dada a maior agregação dos dados.

Em suma, identificou-se que a tendência temporal para o indicador de interrupção do tratamento da tuberculose entre pessoas em situação de rua é influenciada, em especial, pelas seguintes variáveis: sexo, idade, realização do TDO, uso de tabaco, coinfecção tuberculose-HIV e região de notificação. Ainda, observou-se a formação de quatro *clusters*, nos quais estados com piores indicadores sociais tiveram maior porcentagem de descontinuidade do tratamento para tuberculose nessa população.

As evidências direcionam para a necessidade de considerar a influência das características sociais, epidemiológicas, programáticas e territoriais na gestão de políticas, programas e serviços de cuidado às pessoas em situação de rua com a doença. Ainda, é necessário o desenvolvimento de mais pesquisas que avaliem o acesso dessa população a serviços e ações de saúde, com vistas a compreender os obstáculos enfrentados pela PSR durante o seguimento da terapêutica antituberculose.
